# Genesis of Neuronal and Glial Progenitors in the Cerebellar Cortex of Peripuberal and Adult Rabbits

**DOI:** 10.1371/journal.pone.0002366

**Published:** 2008-06-04

**Authors:** Giovanna Ponti, Paolo Peretto, Luca Bonfanti

**Affiliations:** 1 Department of Veterinary Morphophysiology, University of Turin, Grugliasco, Italy; 2 Department of Animal and Human Biology, University of Turin, Turin, Italy; 3 Neuroscience Institute of Turin (NIT), Turin, Italy; 4 National Institute of Neuroscience, Turin, Italy; University of Washington, United States of America

## Abstract

Adult neurogenesis in mammals is restricted to some brain regions, in contrast with other vertebrates in which the genesis of new neurons is more widespread in different areas of the nervous system. In the mammalian cerebellum, neurogenesis is thought to be limited to the early postnatal period, coinciding with end of the granule cell genesis and disappearance of the external granule cell layer (EGL). We recently showed that in the rabbit cerebellum the EGL is replaced by a proliferative layer called ‘subpial layer’ (SPL) which persists beyond puberty on the cerebellar surface. Here we investigated what happens in the cerebellar cortex of peripuberal rabbits by using endogenous and exogenously-administered cell proliferation antigens in association with a cohort of typical markers for neurogenesis. We show that cortical cell progenitors extensively continue to be generated herein. Surprisingly, this neurogenic process continues to a lesser extent in the adult, even in the absence of a proliferative SPL. We describe two populations of newly generated cells, involving neuronal cells and multipolar, glia-like cells. The genesis of neuronal precursors is restricted to the molecular layer, giving rise to cells immunoreactive for GABA, and for the transcription factor Pax2, a marker for GABAergic cerebellar interneuronal precursors of neuroepithelial origin that ascend through the white matter during early postnatal development. The multipolar cells are Map5+, contain Olig2 and Sox2 transcription factors, and are detectable in all cerebellar layers. Some dividing Sox2+ cells are Bergmann glia cells. All the cortical newly generated cells are independent from the SPL and from granule cell genesis, the latter ending before puberty. This study reveals that adult cerebellar neurogenesis can exist in some mammals. Since rabbits have a longer lifespan than rodents, the protracted neurogenesis within its cerebellar parenchyma could be a suitable model for studying adult nervous tissue permissiveness in mammals.

## Introduction

The cerebellum of non-mammalian vertebrates, such as fish, is characterized by striking neurogenesis throughout life [1]. By contrast, the mammalian cerebellum after the early postnatal genesis of granule cells is known as one of the most static structures in the central nervous system (CNS) under the profile of cell renewal. Cerebellar neurons in mammals are generated both centrifugally and centripetally from different sources, at different developmental stages. Purkinje neurons and interneurons originate from the neuroepithelium of the fourth ventricle whereas granule cells come from actively proliferating cell precursors which accumulate in the external granule layer (EGL) after tangential migration from the rhombic lip [2]. The external granule layer (EGL) persists after birth on the cerebellar surface until it provides the granule cell population by radial migration during early postnatal periods whose duration is strictly dependent on the species [3]–[6]. On the other hand, the genesis of cerebellar cortex interneurons occurs by migration through the subjacent white matter and is completed in rodents before the end of granule cell genesis. All neuroepithelium-derived, GABAergic interneurons, including basket, stellate, and Golgi II cell precursors are produced by a common pool of progenitors [7] and express the paired box transcription factor Pax2 [8].

We have recently shown that in the rabbit cerebellum between the fourth and the fifth postnatal week the external granule layer (EGL) is replaced by a proliferative layer which then persists beyond puberty [9]. We called it the subpial layer (SPL) [9] since it is structurally different from the EGL, being characterized by the occurrence of tangential chains of neuroblasts reminiscent of those described in the forebrain subventricular zone (SVZ) [10], [11]. The rabbit subpial layer (SPL) also is transient since it is completely exhausted around the sixth month of postnatal life [9].

Here we analysed the rabbit cerebellar cortex during the period of subpial layer (SPL) occurrence and after its disappearance. We show that a remarkable genesis of cells, mostly neuronal precursors, is detectable within the cerebellar cortical layers at peri-puberal stages, in sharp contrast with the present knowledge in mammals. Besides a residual granule cell genesis which is exhausted very soon, most of these cells differentiate into a population of neuronal cells whose morphology does not fit with any of the known cerebellar cortex neuronal types, and to a lesser extent into a population of Map5+ synantocyte-like progenitors [12], [13]. Surprisingly, by extending our analysis to older animals we found that a remarkable number of cells belonging to both the interneuron and synantocyte types continue to be generated in fully adult animals, even in the absence of a proliferative SPL.

## Results

Animals analysed in the present study will be referred to as postnatal when a typical EGL is present in the cerebellum (from birth to 5 weeks of life), peripuberal when a SPL is detectable on the cerebellar surface (from 2 to 5 months), and adult (1–3 years old rabbits, devoid of cerebellar SPL). Although in rabbits puberty occurs around the fourth month of life, animals aged 2–5 months were considered to be part of a homogeneous group due to the occurrence of the SPL. Most qualitative and quantitative analyses performed on peripuberal rabbits are referred to the three-month old subjects, and differences are reported concerning older animals at specific stages.

### Newly-formed cells with specific morphologies are largely present in the cerebellar cortex of peripuberal rabbits

**Figure 1:**
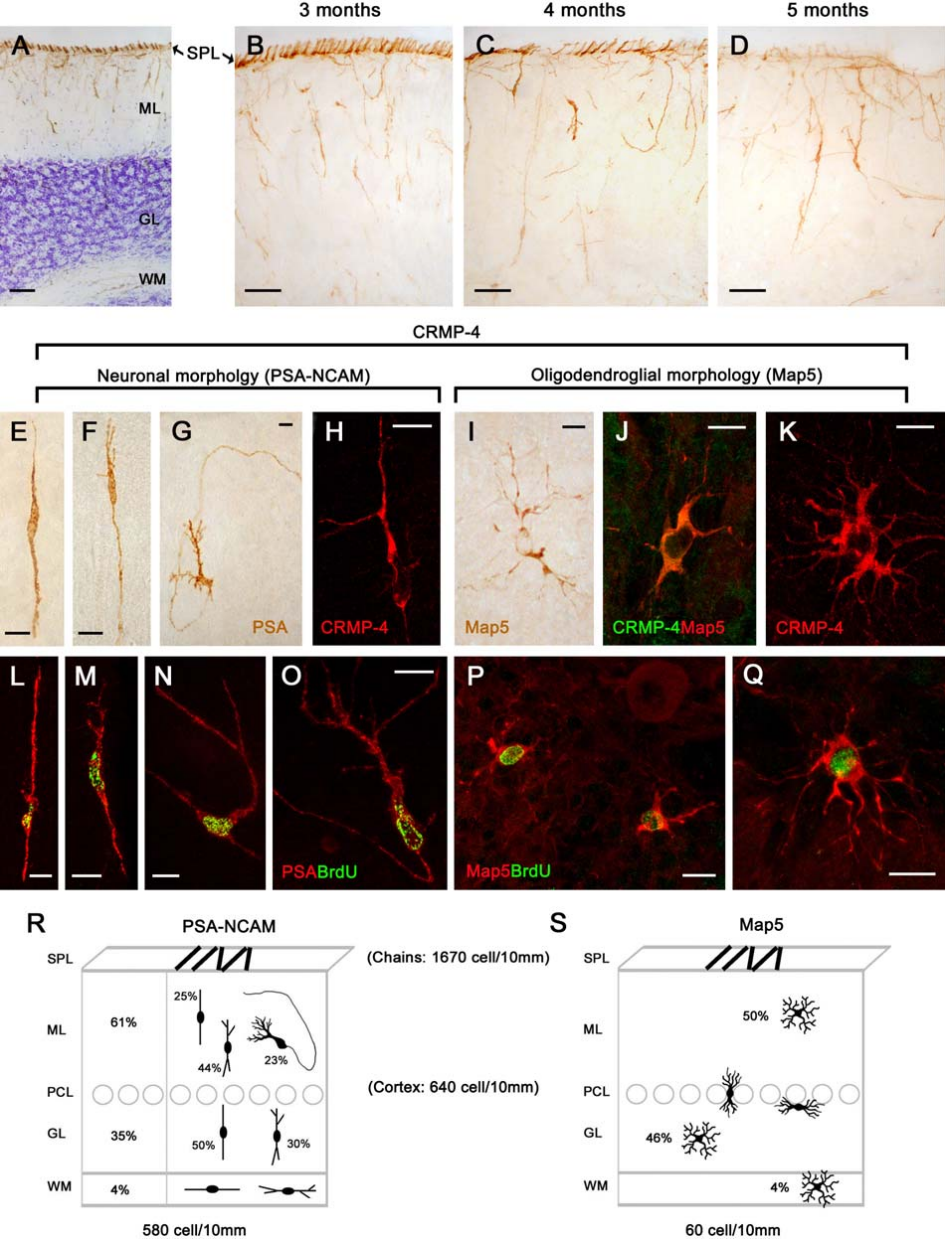
Newly generated cells in the cerebellar cortex of peripuberal rabbits. In addition to the chain of neuroblasts of the subpial layer (SPL; [9]), the peripuberal rabbit cerebellar cortex contains PSA-NCAM+ cells in all layers (A). In parallel with the progressive dilution and disappearance of the SPL, numerous PSA-NCAM+ cells remain detectable in the molecular layer (B–D). PSA-NCAM (E–G) and Map5 (I,J) identify two morphologically-distinct cell populations, both immunoreactive for CRMP-4 (H,J,K). PSA-NCAM+ cells are bipolar- and neuronal-shaped (E–H), whereas Map5+ cells are multipolar (I–K). All these cell types are newly generated, as revealed by BrdU staining (L–Q) at different post-injection survival times (5 [L], 10 [M,N], 15 [O–Q]). Quantitative analysis of the two cell populations in different cerebellar layers (R,S). For PSA-NCAM+ cells in the molecular (ML) and granule layer (GL) different morphologies are also indicated. Bars: A–D, 50 µm; E–Q, 10 µm.

We have previously shown that the cerebellum of peripuberal rabbits is characterized by a proliferative superficial layer (SPL) which contains chains of newly generated neuroblasts (Figure 1A) [9]. In addition, by using markers of cell proliferation the occurrence of many newly generated cells is also visible within the cortical layers, a substantial amount of these cells being still detectable two weeks after their birth [9]. In order to visualize the morphology of these cells, we employed antibodies raised against markers of structural plasticity, namely the polysialylated form of the neural cell adhesion molecule (PSA-NCAM) and the collapsin response mediator protein-4 (CRMP-4) (Figure 1). In spite of the progressive reduction of the SPL during the fourth and fifth month of the rabbit postnatal life, followed by its disappearance at the sixth month, many cells expressing the above-mentioned markers were still detectable in the cortex, particularly in the molecular layer (Figure 1B–D; Table 1). Staining with collapsin response mediator protein-4 (CRMP-4), although fainter than polysialylated form of the neural cell adhesion molecule (PSA-NCAM), revealed cells falling into three main morphological types: bipolar, polarized neuronal-like and multipolar (Figure 1). The polysialylated form of the neural cell adhesion molecule (PSA-NCAM) was detectable in bipolar and polarized, but not in multipolar cells. On the other hand, multipolar cells showed a stellate, highly ramified morphology and were immunoreactive for the cytoskeletal protein Map5 (Map1B; Figure 1I,J) [14], [15].

**Table 1:** Number of cells (cells/10 mm) immunoreactive for PSA-NCAM and Map5 in the peripuberal and adult rabbit cerebellar cortex.

Age (months)	PSA-NCAM (neuronal-like)				Map5 (multipolar)	
	Peripuberal	Adult			Peripuberal	Adult
	3	6	12	36	3	36
**SPL**	1670+/−292.7	0	0	0	0	0
**ML**	360+/−38.9	42.80+/−8.30	12.01+/−5.0	2.44+/−0.7	29.9+/−11.9	2.95+/−1.45
**GL**	200+/−42.1	2.20+/−1.10	1.27+/−0.7	0.14+/−0.1	27.5+/−11.9	2.40+/−1.80
**WM**	20+/−2.9	0	0.07+/−0.1	0	2.3+/−1.4	0.15+/−0.15

T-Test on PSA-NCAM+ and Map5+ cells, in each layer, and at different post-natal age. PSA-NCAM+: 3 month-old vs 6 month-old (ML p = 0,003; GL p = 0,019); 3 month-old vs adult (ML p = 0,003; GL p = 0,018); Map5+: 3 month-old vs adult (ML p = 0,790; GL p = 0,431); T-Test on PSA-NCAM+ and Map5+ cells, in different layers (ML vs GL) at the same post-natal ages; PSA-NCAM+: 3 month-old (p = 0,033); adult (p = 0,178); Map5+: 3 month-old (p = 0,893); adult (p = 0,835).

Quantitative analyses showed that the bipolar/polarized PSA-NCAM+ cell population represents about 90% (580 cell/10 mm +/− 84) on 10% of multipolar, Map5+ cells (60 cell/10 mm +/− 25), with respect to all cortical cells identified by these markers (see Figure 1R,S).

Bipolar cells were mainly radially-oriented, showing the typical morphology of migrating elements, with a leading and a trailing process (Figure 1E). Polarized neuronal cells were observed in the molecular layer with no particular orientation (Figure 1A–D,G,H). They showed a piriform cell body (15–20 µm long), a long, thin axonal-like process with a U-shaped direction remaining within the molecular layer, and one to three thicker, dendritic-like processes on the opposite side of the cell (Figure 1G,H). Quantitative analysis showed that most PSA-NCAM+ cells (61%; 360+/−38,9 cells/10 mm see Figure 1R and Table 1) reside in the molecular layer, the remaining percentage being widespread in the granule cell layer (35%; 200+/−42,1 cell/10 mm) and, to a lesser extent, in the white matter (4%; 20+/−2,9 cells/10 mm) (Figure 1R). In addition to the typical bipolar and neuronal-like morphologies, many intermediate forms characterized by an axonal-like process on one side and some dendritic branches on the opposite side (Figure 1F,M,N) were frequently observed (the relative percentages with respect to the total are given in Figure 1R). The complete set of morphologies, including the neuronal shape, was detectable exclusively within the molecular layer (Figure 1R).

The Map5+ multipolar cells were characterized by a spheric cell body, about 8–10 µm in diameter. These cells showed many, ramified processes reminiscent of the stellate-shaped elements known as synantocytes [12], [13]. Those located in the proximity of Purkinje neurons showed a flattened shape when cut transversely (Figure 1S). In contrast with PSA-NCAM+ cells, they were more homogeneously distributed in both the molecular and granule cell layer (see Figure 1S and Table 1; ML: 29,9 +/− 11,9 cell/10 mm; GL 27,5 +/− 11,9 cell/10 mm) and, to a lesser extent, in the white matter (2,3 +/− 1,4 cell/10 mm).

Morphological observations and countings carried out on both sagittal and coronal sections revealed that PSA-NCAM+ and Map5+ cell populations were homogeneously distributed in all dorsal/ventral and medial/lateral lamellae. To test which among these cells are newly generated we performed double labellings with systemically-administered 5-bromo-2'-deoxyuridine (BrdU) then revealed at different post-injection survival times. As previously described [9], after 2 hours (h) most cell proliferation was observed to occur in the SPL, whereas at subsequent survival times (5–15 days) double stainings of 5-bromo-2'-deoxyuridine (BrdU) with PSA-NCAM and Map5 respectively, clearly revealed that a large population of cells expressing such markers in the peripuberal rabbit cerebellar cortex were newly generated (Figure 1L–Q and Figure S1A).

### Newlyborn elements in the cerebellar cortex of peripuberal rabbits belong to distinct populations of neuronal and glial cell precursors

#### Neuroepithelial-derived interneuronal precursors

**Figure 2:**
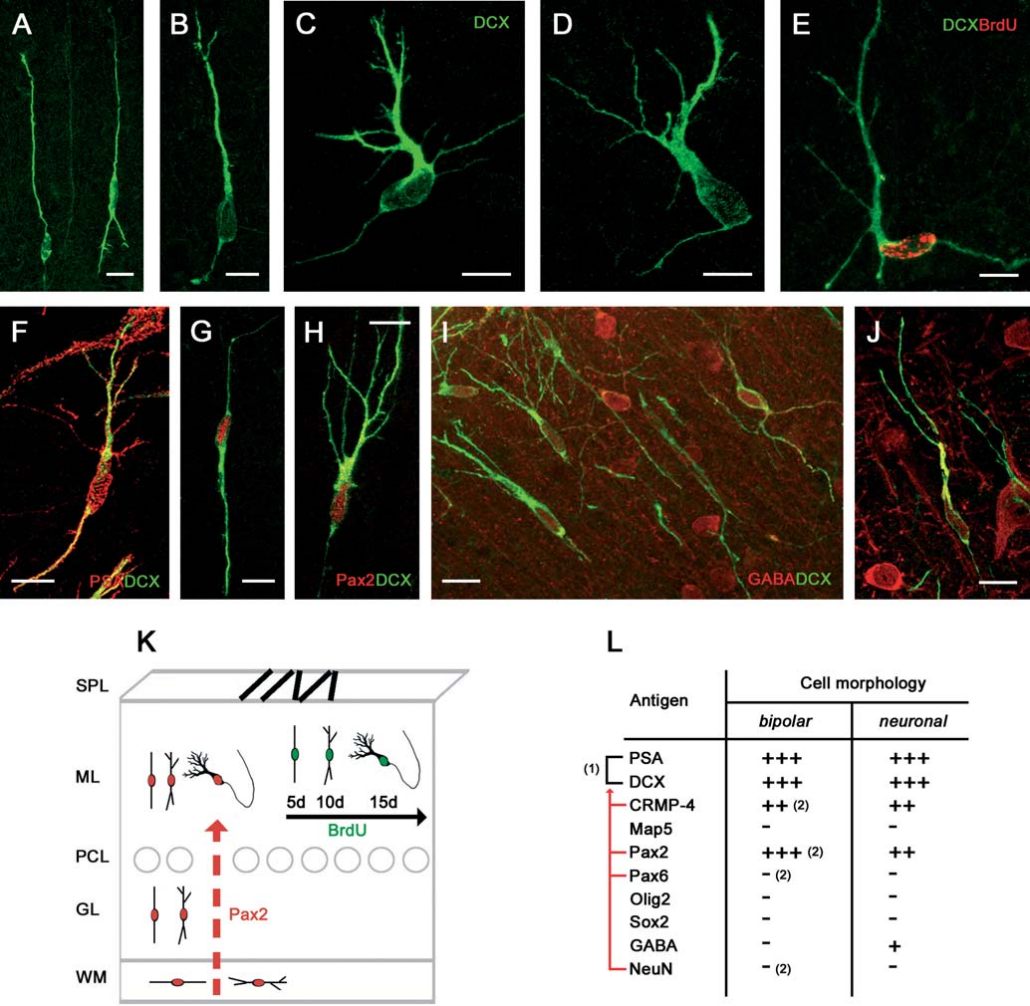
Newly generated, neuronal-shaped cells in the cerebellar cortex of peripuberal rabbits. Bipolar, intermediate, and neuronal-like shaped cells are immunoreactive for the neuronal marker doublecortin (DCX; A–D) and double stained with BrdU (E; 15 days survival post-injection), PSA-NCAM (F), Pax2 (G,H), and GABA (I,J). See also Table in (L); (1), Overlapping populations; red lines, antigens revealing subpopulations of (1); (2), Antigens whose immunoreactivity is present/absent in a subpopulation of bipolar cells (their counterpart being represented in Figure 4L). (K) Model for the newlyborn neuronal cell population: coexpression of PSA-NCAM, DCX, and BrdU (green) in different morphologies reveals a neurogenic sequence within two weeks, and coexpression with the transcription factor Pax2 (red) indicates that these cells are interneuronal progenitors of neuroepithelial origin ascended from the white matter (red arrow). Bars: 10 µm.

The group of PSA-NCAM+ cells including bipolar, intermediate and polarized neuronal morphologies was also immunoreactive for the microtubule binding protein doublecortin (DCX) [16], thus confirming they actually represent a neuronal population (Figure 2A–D). In the rabbit cerebellar cortex, PSA-NCAM and doublecortin (DCX) actually identify an almost overlapping cell population (Figure 2F). Accordingly, in the BrdU/PSA-NCAM double stainings carried out at 5, 10, and 15 days post-injection survival times (Figure 1L–O) the occurrence of bipolar double-labelled cells was more frequent at early survival times, whereas that of more differentiated morphologies (neuronal-like) was detectable at subsequent survival times (see Figure S2A). This indicates that a neurogenic sequence leading to a young neuronal cell type in the molecular layer does occur during a two week temporal window (Figure 2E,K).

In order to further explore the nature and origin of this population of newly generated cells we combined the immunolocalization of the above described markers with different transcription factors involved in the specification of cell progenitors. The transcription factor Pax2, namely a marker for GABAergic cerebellar interneurons of neuroepithelial origin [8;17], was detectable in PSA-NCAM+/DCX+ cells belonging to all morphological types (Figure 2G,H,K). Pax2 staining was generally weaker in ramified cells compared with bipolar ones, thus being probably down-regulated as differentiation proceeds, as confirmed by the incidence of BrdU+/Pax2+ double stainings (see Figure S2B). The same cell population was consistently negative for both the transcription factors Sox2 and Olig2 (Figure 2L). In addition, most polarized neuronal-like cells showed a remarkable staining for the neurotransmitter γ-aminobutyrric acid (GABA, Figure 2I,J), another marker for cerebellar cortex inhibitory interneurons which is concentrated mainly in young neurons before they become terminally differentiated [18]. No γ-aminobutyrric acid (GABA) immunoreactivity was detectable in bipolar-shaped cells, nor in the SPL.

By gathering data of co-expression of cell proliferation markers (at 2 h–15 days survival) with markers for structural plasticity PSA-NCAM, collapsin response mediator protein-4 (CRMP-4), and DCX, the transcription factor Pax2, and the neurotransmitter γ-aminobutyrric acid (GABA) in newlyborn cerebellar cells (summarized in Figure 2K,L), it can be concluded that a subset of GABAergic interneurons are generated within the molecular layer of the peripuberal rabbit cerebellum.

None of the newly generated cells were immunoreactive for specific markers of common cerebellar neuronal cell types, such as calbindin, calretinin, or parvalbumin (Table 2), during the first two weeks after their birth.

**Table 2:** Distribution of other* antigens in newlyborn and non-newlyborn cell populations in the cerebellar cortex of peripuberal and adult rabbits.

Antigen	PSA-NCAM+ or Map5+ newlyborn cell morphologies			BrdU+ nuclei at 30–60 days §	Non-newlyborn cells
	bipolar	neuronal-like	multipolar		
**Parvalbumin**	−	−	−	−	Purkinje, basket, stellate
**Calbindin**	−	−	−	−	Purkinje, Lugaro
**Calretinin**	−	−	−	−	Unipolar brush, Lugaro
**Neurofilaments 20 kD**	−	−	−	−	**Neurons**
**Class III β-tubulin**	−	−	−	−	**Purkinje and Granule cells**
**MAP2**	−	−	−	−	**Purkinje and Granule cells**
**NeuN**	−	−	−	−	**Granule cells**
**α6**	−	−	−	−	Granule cells
**GFAP**	−	−	−	−	Bergmann glia, astrocytes
**Vimentin**	−	−	−	a few astrocytes	Bergmann glia
**S100β**	−	−	−	+ (weak)	Bergmann glia, astrocytes
**BLBP**	−	−	−	+	**Bergmann glia, glial progenitors**
**O4**	−	−	−	−	−

* (antigens not displayed in Tables contained in the Figures).

§ (only in peripuberal animals).

#### Multipolar, oligodendrocytic-like cell precursors (synantocytes)

**Figure 3:**
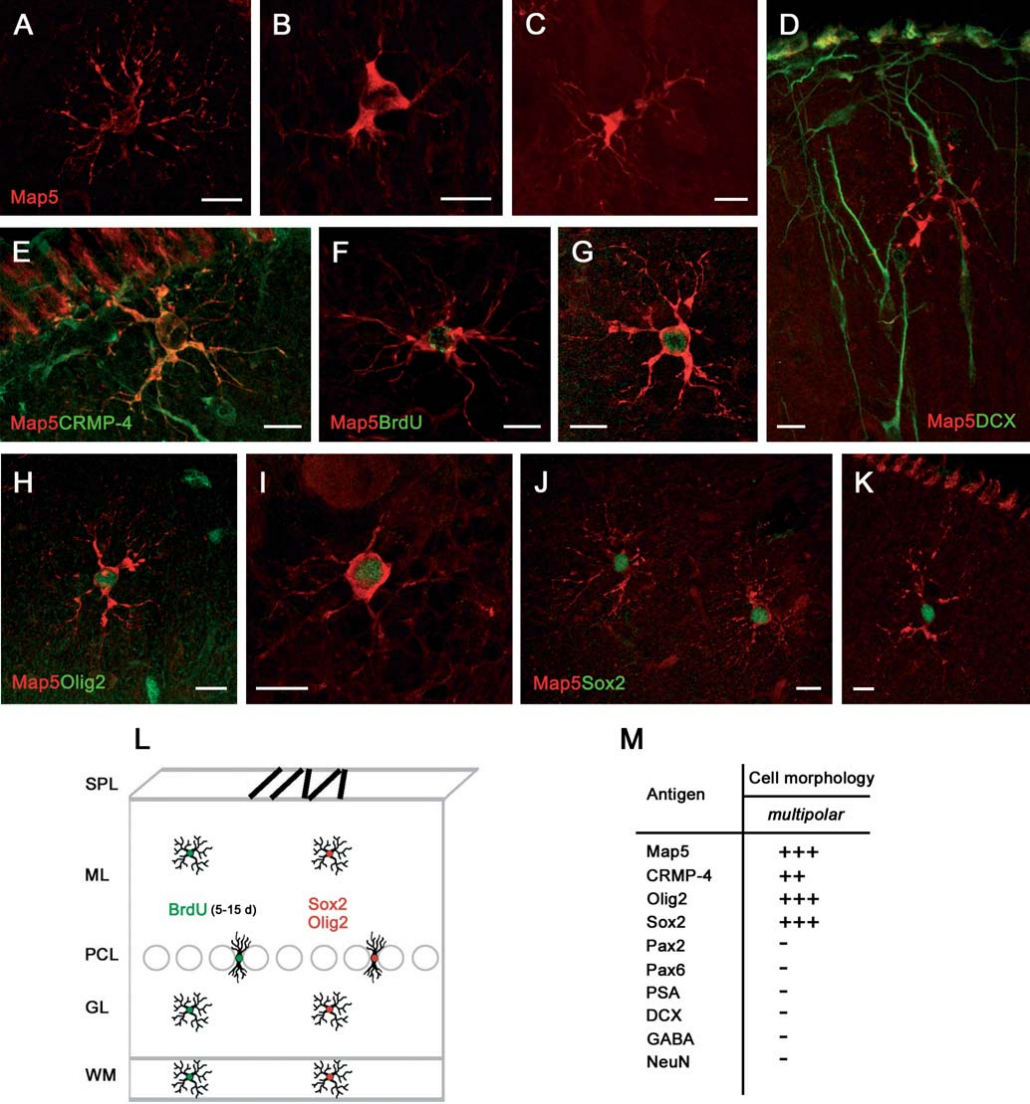
Newly generated, multipolar cells in the cerebellar cortex of peripuberal rabbits. Multipolar cells of different shape are immunoreactive for Map5 in different cerebellar layers (A,D–H,J,K, ML; B,C,I, GL) and CRMP-4 (E), but not DCX (D). These cells are newly generated, as they are marked with BrdU at 15 days survival post-injection (F,G). They also express the transcription factors Olig2 (H,I) and Sox2 (J,K). See also Table in (M). (L) Model for the newlyborn Map5+ cell population: the cells are homogeneously distributed in all cerebellar layers. Bars: 10 µm.

The multipolar, Map5+ cells (Figure 3A–C) were immunoreactive for CRMP-4 but not PSA-NCAM, DCX, or GFAP (Figures 1I–K and 3D,M; Table 2). They were consistently double-stained for both the transcription factors Sox2 and Olig2 [19;20] (Figure 3H–K,L) but not for Pax2 (Figure 3M). Thus, the distribution of a combination of cell markers and transcription factors suggests that the PSA-NCAM+/DCX+ cells and the Map5+ cells actually represent two distinct populations, the former being far more numerous than the latter, with a 9/1 ratio in the three-month old animals. Student's Test-T revealed that the number of PSA-NCAM+ cells in 3 month-old rabbits is significantly higher than the number of Map5+ cells (ML p = 0,003 GL p = 0,019; Figure 1R,S and Table 1).

Using BrdU/Map5 double stainings (Figure 3F,G) many Map5+ multipolar cells (Figure S1A) were found to be generated, starting from the second week survival time after the injection of the DNA marker. No particular spatio-temporal pattern was detectable in these newlyborn cells by considering different morphologies at different survival times and in different cerebellar layers (Figure 3L). Unlike PSA-NCAM+/DCX+ cells, none of the Map5+ cells were found to be double-stained for BrdU at early (1 to 5 days) post-injection survival times, thus indicating that Map5 starts to be expressed later (see Figure S1A,B).

The immunocytochemical characterization of multipolar cells with transcription factors, markers of cell proliferation and neurogenesis, indicated that in the peripuberal rabbit cerebellar cortex, in addition to molecular layer interneurons another population of newly generated cells involves glial-like cell progenitors. These cells are reminiscent of synantocytes [13], a recently described cell type sharing a morphology of protoplasmic astrocytes in vivo and antigenic features of oligodendrocyte precursor cells in vitro. This cell category could not be further identified by expression of Ng2 antigen [12], since none of the antibodies tested were working on the rabbit tissue (see Table 3). The absence of Map5, Olig2, and Sox2 immunoreactivity in bipolar-shaped cells does suggest that the newlyborn multipolar cells come from local progenitors in each cerebellar layer.

**Table 3:** Antibodies used in this study.

Antigen	Dilution	Antibody (clone)	Sp.	rb	Source	Cat. N°
**Proliferation markers**						
Ki67	1∶300	(mono) MIB-1	m	+	Dako	7240
BrdU	1∶600	(mono) BU1/75	rat	+	Harlan	MAS250c
**Markers of stem/progenitor cells**						
Sox2	1∶1500	(poly)	rb	+	Chemicon	AB5603
	1∶500	(poly)	g	+	Chemicon	sc1739
BLBP	1∶2000	(poly)	rb	+	Chemicon	AB9558
**Neuronal markers**						
Doublecortin (C18)	1∶750	(poly)	g	+	Santa Cruz	sc8066
Class III β-tubulin (TUJ1)	1∶1000	(poly)	rb	+	Covance	PRB435P
MAP5	1∶5000	(mono) AA6	m	+	Chemicon	MAB366
MAP2	1∶500	(mono)AP20	m	+	Chemicon	MAB3418
NeuN	1∶400	(mono)A60	m	+	Chemicon	MAB377
Hu C/D	1∶200	(mono)16A11	m	+	Molecular Probes	A21271
CRMP4/Tuc-4	1∶500	(poly)	rb	+	Chemicon	AB5454
GABA	1∶5000	(poly)	rb	+	Sigma	A2052
Pax2	1∶200	(poly)	rb	+	Zymed	71–6000
Pax6	1∶800	(poly)	rb	+	Covance	PRB278P
Calbindin	1∶1500	(mono)	m	+	Swant	300
Parvalbumin	1∶7000	(mono) PARV19	m	+	Sigma	P3088
Calretinin	1∶2500	(poly)	rb	+	Swant	7699/4
α6 GABA_A_R	1∶100	(poly)	rb	+	Chemicon	AB5453
Neurofilament 200 kD	1∶400	(mono) NE14	m	+	Sigma	N5489
**Glial markers**						
Vimentin	1∶800	(mono) V9	m	+	Dako	M0725
GFAP	1∶1000	(poly)	rb	+	Dako	Z0334
Ng2	1∶250	(poly)	rb	−	Chemicon	AB5320
	1∶300	(mono)	m	−	Chemicon	MAB5384
	1∶100	(mono) 132.38	m	−	Sigma	N8912
	1∶10000	(mono) 132.38	m	−	Upstate	05–710
O4	1∶200	(mono) 81	m	+	Chemicon	MAB345
S100β	1∶10000	(poly)	rb	+	Swant	37
Olig2	1∶1500	(poly)	rb	+	Chemicon	AB9610
	1∶400	(poly)	g	+	R&D Systems	AF2418
**Migration markers**						
PSA-NCAM	1∶3500	(mono)2–2B	m	+	AbCys	AbC0019
TAG-1	1∶800	(mono)4D7	m	+	DSHB	TAG-1

Sp., species; m, mouse; rb, rabbit; g, goat; + works on rabbit tissue; − does not work on rabbit tissue.

None of the newly generated cells were clearly double-stained for the glial markers GFAP or O4 (Table 2). Also double stainings between BrdU and vimentin or S100β, did not yield clear coexpression in identifiable glial cell types, due to the faint expression (S100β) or prevalent localization of these markers in cell processes (vimentin) rather than in the cell body (not shown).

#### Residual granule cell precursors

**Figure 4:**
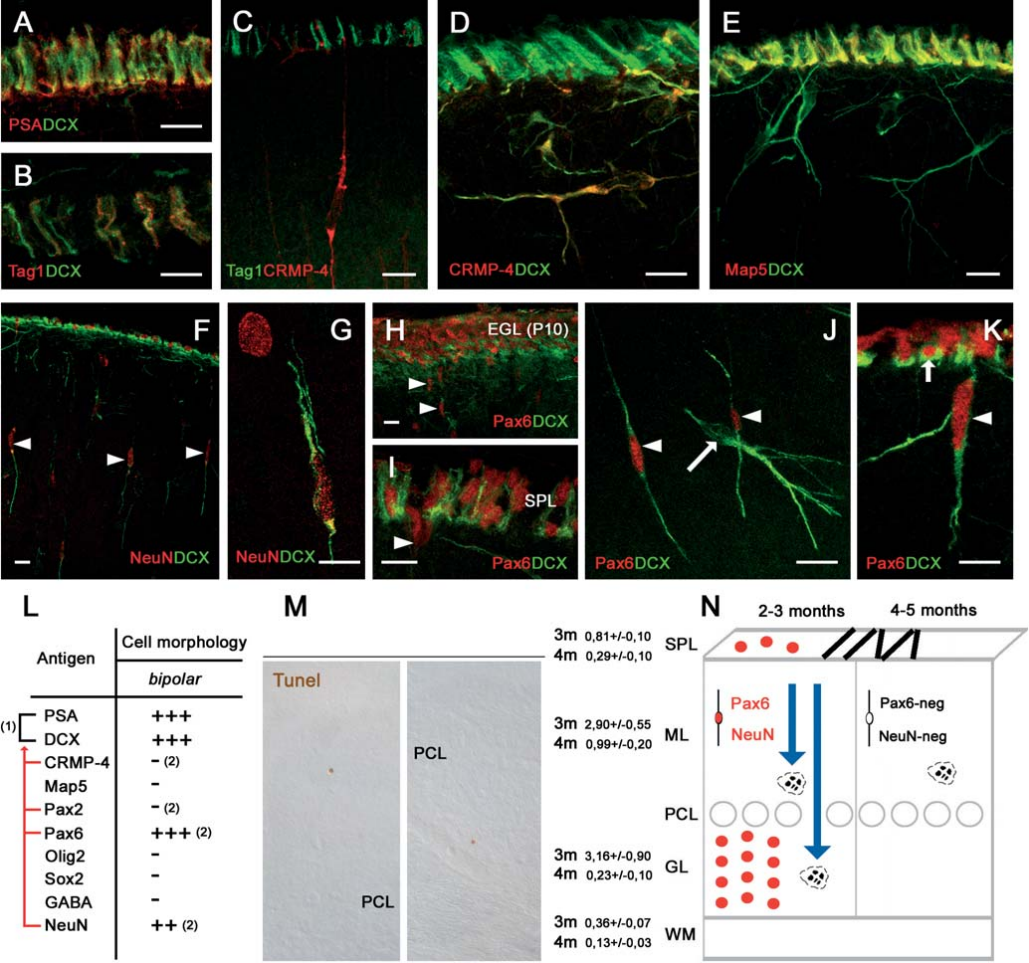
Residual granule cell precursors in the cerebellar cortex of peripuberal rabbits. Neuroblasts forming chains in the SPL are immunoreactive for PSA-NCAM (A), DCX (A,B,E), and Map5 (E), partially for Tag1 (B,C) and NeuN (F), but not for CRMP-4 (D). NeuN (F,G) and Pax6 (H–K) reveal residual granule cell precursors both in the SPL and in the ML, but they are not present in all DCX+ cells (J). See also Table in (L); (1), Overlapping populations; red lines, antigens revealing subpopulations of (1). (2), Antigens whose immunoreactivity is present/absent in a subpopulation of bipolar cells (their counterpart being represented in Figure 2L). (M), Cell death revealed with Tunel technique is reduced in the shift from the third to the fourth month of age (3 m, 4 m), suggesting that granule cell genesis is coming to an end. Student's T-test revealed a significant decrease of the number of tunel labelled cells in each layer (SPL p = 0,010; ML p = 0,005; GL p = 0,004 WM p = 0,013).This is confirmed by the fact that Pax6 and NeuN are no more detectable after the fourth postnatal month (N). Bars: 10 µm.

Bipolar-shaped cells in the cerebellar cortex of peripuberal rabbits represent about 30% of all cells detectable with the above-mentioned markers (Figure 1R). As described above, a consistent part of these cells are Pax2+/PSA-NCAM+/DCX+ and belong to the population of newly generated neuronal progenitors (Figure 2K). A subpopulation of the PSA-NCAM+/DCX+, but Pax2-negative bipolar cells were immunoreactive for NeuN [21] (Figure 4F,G) and Pax6 [22], [23] (Figure 4J,K), two markers for cerebellar granule cell precursors (Figure 4H). The detection of Pax6+ and NeuN+ bipolar cells was restricted to the second and third postnatal months, being absent starting from the fourth month and so forth at all later stages (Figure 4N). In addition, Pax6 immunoreactivity was never observed in neuronal-shaped cells (Figure 4J). This indicates that some granule cell precursors continue to migrate from the SPL to the cortical layers during the early phases after the shift from EGL to SPL. Then, this protracted descent of granule cell precursors is exhausted before puberty. The late granule cell precursors seem not to differentiate into mature granule cells, since no immunoreactivity for the GABA_A_ receptor subunit α6 [24] was detectable in newlyborn cells (not shown). In addition, consistent apoptotic cell death was present in both layers of the cerebellar cortex in three month-old animals (Figure 4M), whereas it is significantly reduced in the four month-old rabbits, particularly in the granule cell layer (Figure 4M; summarized in Figure 4N).

The occurrence of a residual descent of granule cell precursors raises questions about the persistence of SPL chains of tangentially-oriented cells. These chains are progressively reduced in number from the second to the fifth month, then disappearing from the cerebellar surface during the sixth month. The early neuronal markers doublecortin (Figure 4A,B), NeuN (Figure 4F), class III β-tubulin [25], and HuC/D protein (not shown) were detected in subpial chains, thus confirming they are formed by neuroblasts (see also [9]). Yet, not all antigens detectable in the SPL were co-expressed in cortical cells (see Figure 4). For instance, CRMP-4 although present in newlyborn neurons of the molecular layer was absent in SPL chains (Figure 4C,D). By contrast, the neuronal cell adhesion molecule TAG-1 [26] was detectable in a subset of these chains (Figure 4B) but not in cortical cells (Figure 4C). Similarly, Map5 (Map1B) which is involved in the control of microtubule organization in both neuronal and glial progenitors [14], [15], was present in all SPL chains but absent in DCX+ newlyborn neurons (Figure 4E). These data, in parallel with the neuroepithelial origin of the Pax2+ interneuronal population (see above), suggest that the transient SPL does contain the last granule cell precursors which mostly fail to reach their final destination in the granule cell layer.

#### Newly generated cells followed for survival times longer than two weeks

**Figure 5:**
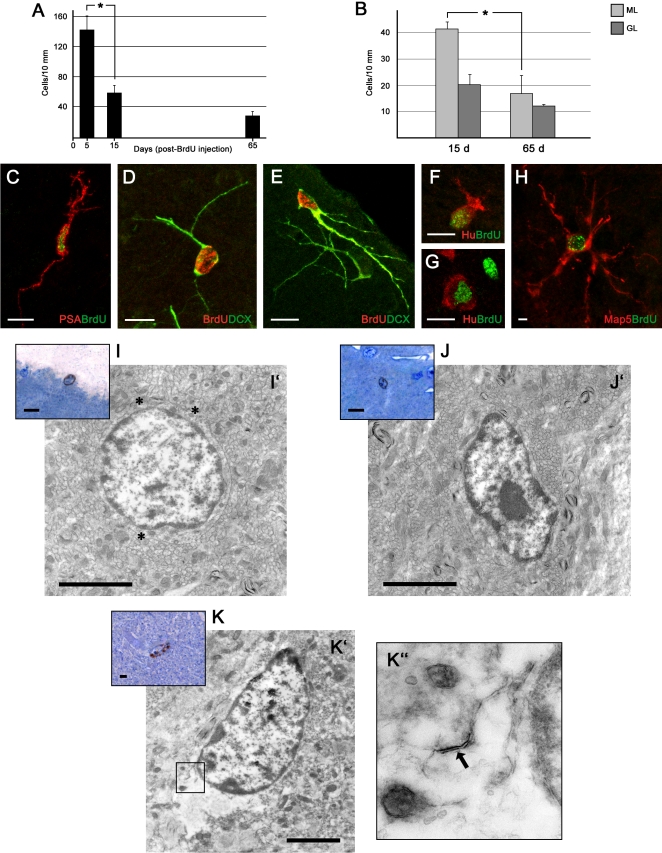
Newly generated cells 1–2 months after their genesis in the cerebellar cortex of peripuberal rabbits. Countings of BrdU+ cells in the cerebellar cortex at 5, 15, and 65 days after injection (A) reveal a marked drop during the first two weeks followed by a relative stabilization during the subsequent months (F = 19,01, p = 0,001). Post-hoc comparison revealed a decrease in the number of BrdU+ cells between 5 and 15 days. (B) The slight decrease observed at long term survival mainly affects cells in the molecular layer (ML). Student's T-test revealed that the decrease of the number of cells between 15 and 65 days of survival in the ML shows a trend (p = 0,09) while in the GL it is not significant (p = 0,45). C–H, BrdU-stained cells visualized 1 month (C–G) and 2 months (H) after the last of 5 injections in association with different neuronal (D–G) and structural plasticity (A,H) markers. (GL) granule cell layer. I–K, Ultrastructural analysis of BrdU+ cells 2 months after systemic injection of the DNA marker. After identification of the marked nuclei in semithin sections of the molecular layer (I,J,K, color insets), the same cells localized at the electron microscope in ultrathin sections (I',J',K') reveal features of a synantocyte (I'), showing a round cell soma and three initial, multipolar processes (asterisks), and two polarized neurons with an oval cell soma (J',K'). K'', inset showing an higher magnification of an axo-somatic synapse (arrow). Bars: A–F, 10 µm; I,I',J,J',K,K', 3 µm; (the cells in ultrastructural photographs seem smaller than in confocal images since the sections are not cut through their main diameter).

Cell countings of BrdU+ cells extended up to two months survival indicated that most of the cells still alive in the cortex two weeks after their birth, actually survive for at least two months, in contrast with a remarkable drop occurring during the first two weeks [9] (Figure 5A,B).

Although some newlyborn cells were still detectable in association with PSA-NCAM (Figure 5C), DCX (Figure 5D,E), and Hu protein (Figure 5F,G) up to 30 days since their birth, after this period most of the BrdU+ nuclei were not identifiable through double stainings with cytoplasmic or membrane-bound markers that could reveal a specific cell morphology (Figure S1B). Unlike neuronal precursor cells, the Map5+ cells started to express the cytoplasmic marker two weeks after their genesis. Then, the Map5 staining was still detectable until 65 days indicating that these cells maintain their multipolar morphology for months (Figure 5H and Figure S1B). Accordingly, also the number of BrdU+/Sox2+ double labelled cells was very high between two weeks and two months after their genesis (Figure S2C).

No more GABA staining was detectable in newlyborn cells at long survival times, this neurotransmitter being downregulated as cell differentiation occurs [18]. In addition, none of the newly generated cells aged more than one month were immunoreactive for the interneuronal markers calbindin, parvalbumin, calretinin (see Table 2).

Some BrdU/vimentin and BrdU/S100β double stainings (not shown) were observed within the granule cell layer and Bergmann glial cell layer (Table 2). Most of these cells were not clearly identifiable as to their morphology. This indicates that also astrocytes could be generated at least within the granule cell layer, and suggests that some Bergmann glial cells might proliferate (see below).

Finally, rare Isolectin B4+ (IlB4) microglial cells (3,16 +/− 1,84%) that had incorporated BrdU were detectable at 65 days post-injection survival (Figure S1D).

Thus, it appears that a large amount of newly generated neuronal precursors cannot be visualized beyond the first month after their birth, due to a lack of specific cytoplasmic/membrane-bound markers (Figure S1B). For these reasons, in order to visualize the newlyborn cells at longer survival times, we carried out an ultrastructural study on BrdU-treated rabbits that were left to survive 60 days after the first and 45 days after the last injection of the marker. After visualization of the BrdU+ nuclei by peroxidase staining, small pieces of the cerebellar molecular layer containing these cells were resin-embedded and processed for electron microscopy. The marked nuclei were easily identified in semithin sections (Figure 5I–K) and subsequently photographed in ultrastructure. Cell types corresponding to the size and shape of synantocytes (Figure 5I') and neurons (Figure 5J',K') were found, thus showing that elements belonging to both cell populations are still in place after two months. In two cells out of 8 cells analyzed, the occurrence of rare axo-somatic synaptic contacts was observed (Figure 5K'').

### Newly generated neuronal and glial cell progenitors in the cerebellum of adult rabbits

**Figure 6:**
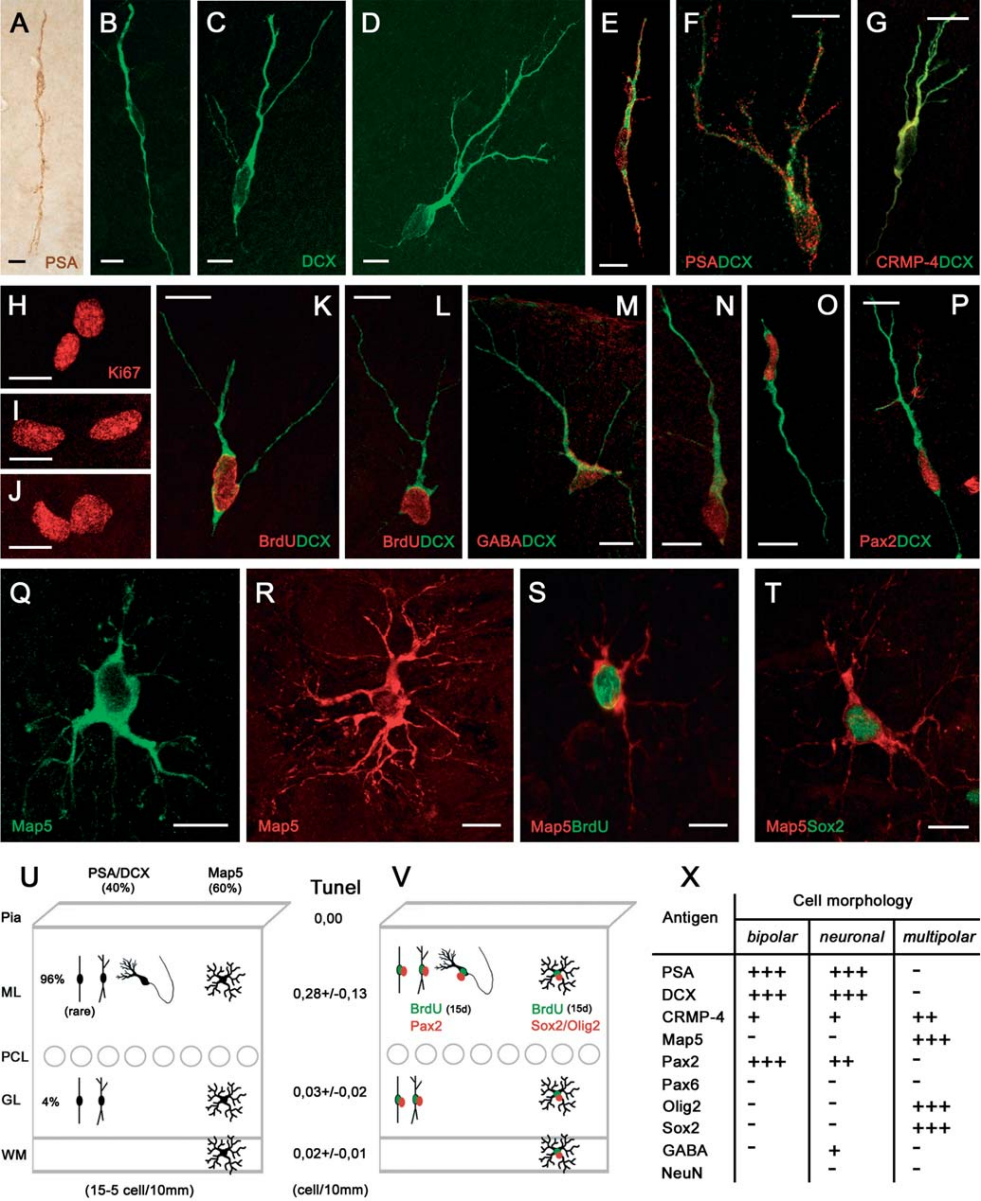
Newly generated cells in the cerebellar cortex of the adult rabbit. All morphological cell types, expressing and coexpressing the same markers as in the peripuberal rabbit cerebellum are detectable in the adult (1 and 3 years; A–G,Q,R). Cell proliferation is detectable in both cortical layers, as showed by Ki67 staining (H–J), and newly generated neuronal (K,L) and multipolar, glial-like cells (S) are visible after BrdU staining at 15 days survival. Also in the adult, the newlyborn neuronal cells do contain GABA (M,N) and Pax2 (O,P), as well as the multipolar cells are immunoreactive for Sox2 (T). See also Table in (X). U, Absolute and relative amounts of PSA-NCAM+ and Map5+ cells in the adult cerebellum. V, Model for the newlyborn PSA-NCAM+ and Map5+ cell populations in the adult. Bars: 10 µm.

The cerebellum of adult rabbits was completely devoid of SPL. By contrast, some Ki67+ nuclei were still detectable in the cortex (Figure 6H–J). The Ki67+ nuclei were frequently detected as ‘doublets’ (Figure 6H–J), indicating their feature of daughter cell progeny of a recent mitotic event [27]. The occurrence of newly generated cortical cells in these animals was confirmed by detection of BrdU after 15 day survival (Figure 6K,L,S). The newlyborn cells were present in both cortical layers, more frequently located close to the Purkinje cell layer, on both sides.

**Figure 7:**
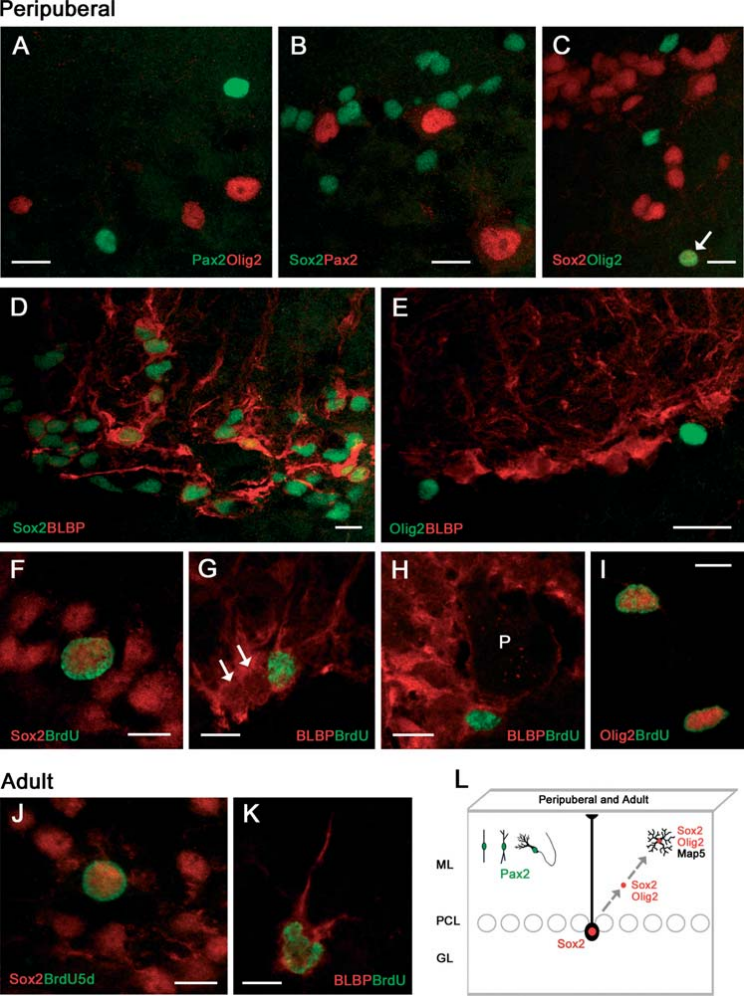
Relationships among transcription factors in newly generated neuronal and glial-like cell populations. Both Olig2 and Sox2 are never coexpressed with Pax2 (A,B), whereas some Sox2+ nuclei also contain Olig2 (C, arrow). Virtually all BLBP+ Bergmann glia cells contain Sox2 (D) but not Olig2 (E). Some large Sox2+ nuclei in the Purkinje cell layer do incorporate BrdU at 1–5 days postinjection survival (F). Incorporation of BrdU is also detectable in some Bergmann glia cells (G; two immuno-negative Bergmann glial cells are indicated by arrows), that retain the marker for at least two months (H). P, Purkinje neuron (immuno-negative). Also Olig2+ cells can incorporate BrdU at 1–5 days postinjection survival (I). (J,K), Proliferating Sox2+ nuclei and BLBP+ Bergmann glia cells in the Purkinje cell layer of the adult rabbit. (L), Schematic summary: Pax2+ neuronal precursors and multipolar glial-like cell precursors generated in the peripuberal and adult rabbit cerebellar cortex belong to two distinct populations. Sox2+, dividing Bergmann glia could generate Sox2+/Olig2+ precursors that then express Map5 in multipolar cells. Bars: 10 µm.

By performing the same immunocytochemical analyses carried out in the peripuberal animals, similar PSA-NCAM+/DCX+ neuronal-shaped cells and Map5+ synantocyte-like cells were detected in the adult cerebellum (Figure 6). Both cell types showed the same morphology, antigenic features, and layer distribution described in the three-month old cerebellar cortex. Nevertheless statistical analysis revealed that in the adult the PSA-NCAM+/DCX+ and Map5+ cells are equlibrated (ML p = 0,790; GL p = 0,431) Accordingly, the PSA-NCAM+/DCX+ neuronal cells were immunoreactive for GABA (Figure 6M,N). By contrast, some differences concerned the absence of PSA-NCAM+/DCX+ cells within the white matter of adult cerebella, and the very rare occurrence of bipolar-shaped cells in the cortex (summarized in Figures 6U and 7B).

Double stainings with DCX or Map5 and BrdU at 15 day survival after the injection revealed that both cell populations in the adult cerebellar cortex were newly generated (Figure 6L,M,S). Similarly to what observed in peripuberal rabbits, the PSA-NCAM+ and DCX+ bipolar and neuronal cells co-expressed the two markers (Figure 6E,F), and most of them were also immunoreactive for CRMP4 (Figure 6G) and the transcription factor Pax2 (Figure 6O,P). Accordingly, the Map5+ stellate-shaped cells co-expressed Sox2 (Figure 5T) and Olig2 (not shown). On the other hand, no Pax6+ cell could be detected in the adult cerebellar cortex.

Countings carried out on adult animals (see Table 1) indicated that a further reduction in the number of newly generated cerebellar progenitor cells does occur with respect to the six-month old rabbit, with a ratio of 10/1 at the age of three years (Figure 6U). About one half of these cells are neuronal progenitors that are mostly localized in the molecular layer (see percentages in Figure 6U).

### Relationships among cell proliferation markers, transcription factors, and BLBP in newly generated cells of the postnatal and adult rabbit cerebellum

In order to get more insights about the origin of the two populations of newly generated cortical cells, cross double stainings among different transcription factors were carried out (Figure 7). These experiments clearly showed that both Olig2 and Sox2 were never coexpressed with Pax2 (Figure 7A,B), whereas some Sox2+ nuclei also contained Olig2 (Figure 7C). These results support the hypothesis that the Pax2+ neuronal cell precursors belong to a distinct lineage with respect to the multipolar, glia-like cells.

Since it is known in mice that Sox2 is expressed in the nuclei of Bergmann glia [28] we used the marker Brain Lipid Binding Protein (BLBP) to visualize cell bodies of Bergmann glia, in order to further investigate this radial glia-derived cerebellar cell type in rabbits. Double labellings showed that virtually all Bergmann glia cells contain Sox2 (Figure 7D) but not Olig2 (Figure 7E). In addition, some large Sox2+ nuclei in the Purkinje cell layer did incorporate BrdU at 1–5 days postinjection survival (Figure 7F and Figure S1C), and incorporation of BrdU was also detectable in some Bergmann glia cells (Figure 7G). These latter were able to retain the proliferation marker for at least two months (Figure 7H). Also Olig2+ cells can incorporate BrdU at 1–5 days postinjection survival (Figure 7I).

Proliferating Sox2+ nuclei and Brain Lipid Binding Protein (BLBP)+ Bergmann glia cells were also detectable in the Purkinje cell layer of adult rabbits (Figure 7J,K).

On the whole, these results indicate that Sox2+ dividing Bergmann glia could generate cell precursors that in the following days will express both Sox2 and Olig2, and then Map5, thus becoming visible as multipolar cells (summarized in Figure 7L). Furthermore, they confirm that Pax2+ neuronal precursors and multipolar glial-like cell precursors generated in the peripuberal and adult rabbit cerebellar cortex belong to two distinct populations.

## Discussion

Persistent neurogenesis is a phylogenetically highly conserved feature in the animal world [29]. A general rule views the genesis of new neurons to be rather widespread in invertebrates and in non-mammalian vertebrates such as fish, reptiles and birds, but more restricted to specific brain areas in mammals [30]. In this animal class adult neurogenesis is consistently present only within two brain areas: the forebrain SVZ and the hippocampal dentate gyrus [30]–[33]. Yet, recent comparative studies have started to show that important differences also exist among mammals, concerning the topographical location of neurogenesis, the internal arrangement of adult neurogenic sites, and the mode/direction of cell migration (reviewed in [33]) [34]. For instance, striking differences have been found to characterize the rabbit brain, in the form of parenchymal chains leaving the SVZ for the mature parenchyma in young animals [35], as well as remarkable genesis of interneurons within the adult caudate nucleus [36].

Unlike the brain, the mammalian cerebellum is considered completely uncapable of cell renewal after the end of the postnatal granule cell genesis. Thus, plasticity in this CNS region is thought to be limited to synaptic rearrangement of pre-existing circuits [37]. In contrast, we recently demonstrated the existence of a proliferative SPL on the rabbit cerebellar surface, which persists beyond puberty (from the second to the fifth month of life) as an extension of the EGL, although with different structural features [9]. Since a great number of newlyborn elements are also detectable within the cerebellar cortical layers of these rabbits, here, after investigating the nature, distribution and fate of the newly generated elements in young animals we show that two populations of neuronal and glial progenitors continue to be generated during adulthood.

### Protracted genesis of neuronal and glial progenitor cells in the peripuberal rabbit cerebellum

We started from previous cell countings of BrdU+ nuclei carried out in three-month old animals, indicating that a huge amount of cells are newly generated within the cortex, about 1/3 of them surviving during the first two weeks after the injection of the marker [9] (Figure 5A). In order to reveal the nature of newlyborn cortical elements, the cells containing BrdU+ nuclei were visualized in double stainings with the most typical developmental markers PSA-NCAM, DCX, CRMP-4, and Map5. All these molecules are implicated in dynamic cellular events both during developmental and adult neurogenesis, and allow the visualization of the whole cell morphology, including cell body and processes [11], [16], [38], [39].

**Figure 8:**
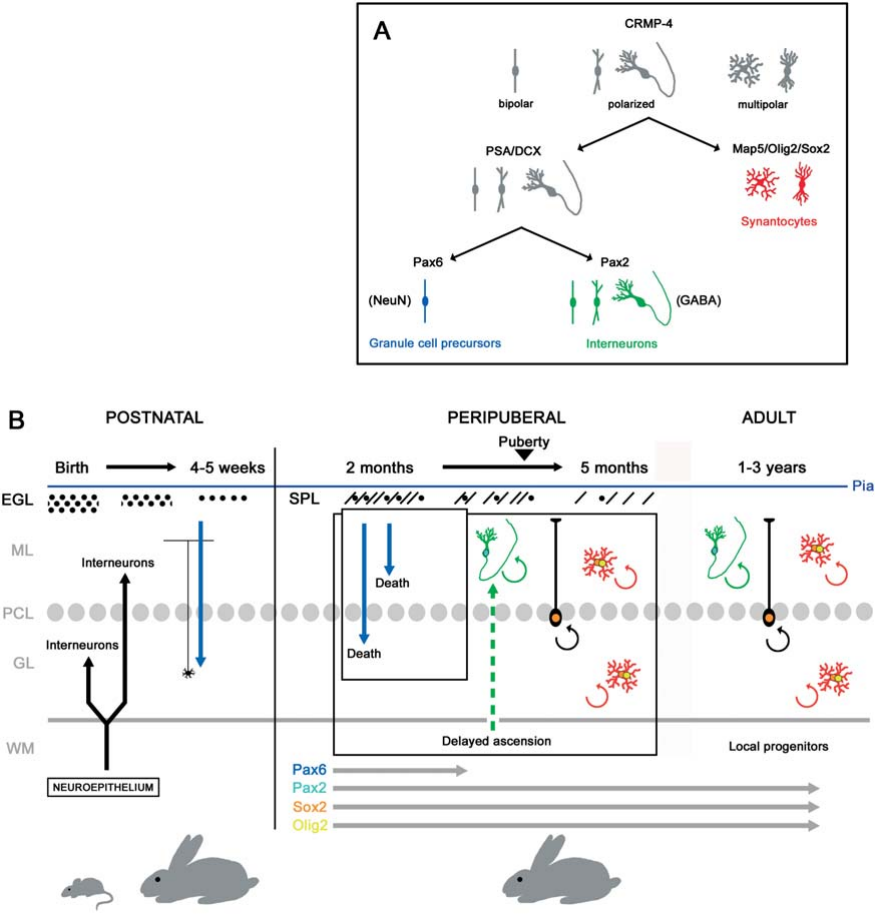
Model for protracted cell genesis in the cerebellum of the peripuberal and adult rabbit. A, Summary of antigens distribution and morpholgies associated with different populations of newlyborn cells in the rabbit cerebellar cortex. B, Protracted neurogenesis in the postnatal/peripuberal cerebellum and persistent neurogenesis in the adult. All processes represented at the right of the vertical line are typical of rabbits.

A great number of cells expressing and co-expressing such markers were detectable in the cerebellum of peripuberal and puberal rabbits, in overt contrast with their complete absence in the postnatal cerebellum of rodents [9], [40], [41]. Different molecules were associated with specific morphologies: PSA-NCAM and DCX, which are known to be strongly expressed in newly generated neuronal precursors [11], [16], [38], were found in bipolar and neuronal-shaped cells, whereas Map5 was found in multipolar cells reminiscent of an intermediate astrocytic/oligodendrocytic shape (Figure 8A).

The fact that polarized neuronal cells and multipolar cells actually belong to two distinct populations of newlyborn cerebellar cortical cells was confirmed by their selective expression of distinct nuclear transcription factors: the PSA-NCAM+/DCX+ cells (Figure 2G,H,K) were immunoreactive for Pax2 [8], [17], and were consistently negative for both Sox2 and Olig2 (Figures 2 and 7A). By contrast, all MAP5+ cells were Olig2+, Sox2+, and Pax2-negative (Figures 3 and 7A).

### Interneurons and synantocyte/polydendrocyte-like cells are generated in the cerebellum of peripuberal rabbits

The neuronal cells displayed shapes reminiscent of the differentiative sequence previously described in brain neurogenic sites, involving bipolar cells that subsequently emit cell processes and give rise to ramified neurons [42]. These cells expressed the anti-adhesive, membrane-bound molecule PSA-NCAM [11], and the two cytosolic phosphoproteins CRMP-4 and DCX, both implicated in the regulation of the actin cytoskeleton in motile cells [39], [43]. All these molecules are strictly associated to adult-generated neuronal precursors in the hippocampus and SVZ, being required for their migration and structural plasticity [11], [16], [38]. In addition, the PSA-NCAM+/DCX+ neuronal cells expressed in their nuclei the marker for GABAergic cerebellar interneurons Pax2. This transcription factor specifically identifies the neuronal precursors of neuroepithelial origin ascending through the cerebellar white matter to colonize the cerebellar cortex during the early postnatal period [7], [8], [17]. The facts that these cells are restricted to the molecular layer and are immunoreactive for GABA confirm that they are independent from granule cell genesis (Figures 5H; see below). Indeed, the existence of a subpopulation of PSA-NCAM+/DCX+ newlyborn cells with bipolar morphology and antigenic features of granule cell precursors (NeuN and Pax6) was restricted to the third-fourth postnatal month (Figure 8B). A substantial genesis of granule cells in peripuberal rabbit cerebella can also be excluded for several reasons: i) most newly generated cells belonging to the neuronal population were found within the molecular layer, and those displaying a differentiated neuronal morphology and GABA-ergic expression were found exclusively in this layer; ii) the subpopulation of Pax6+ cells was no more detectable after the fourth postnatal month, in contrast with the other two cell populations that persisted up to adulthood (see below). In addition, even at earlier stages these cells did not give raise to ectopic granules which have been described in the molecular layer of the rabbit [44], since they were never seen to form clusters. Finally, the identity of newly generated cells with terminally differentiated granule cells of the granule cell layer seems to be excluded by the absence of labelling with the cerebellar granule cell marker GABA_A_ receptor subunit α6 [45] in BrdU+ cells at different survival times, both in the molecular and the granular layers.

A second group of newlyborn elements that were detectable beyond puberty involved multipolar cells immunoreactive for Map5, Sox2, and Olig2. In the three-month old rabbits these cells represented a small population (about 1/9 with respect to the newly generated neuronal cells), whereas their relative amount increased in the adult, reaching almost half of the total. The microtubule-associated protein Map5 (MAP1B) has been described in cells of both the neuronal and glial lineage. It is believed to play an important role in the outgrowth of neurites from neurons [46] and it is present in proliferating neuroblasts of the embryonic mouse telencephalic ventricular zone [47]. In addition, although negligible in astrocytes, its expression precedes the development of the mature oligodendrocyte phenotype and interactions between microtubules and Map5 has been suggested to have a role in the formation and stabilization of myelin-forming processes [48]. In general, the expression of Map5 and its mRNA remains at relatively high level in neurogenic/structurally plastic regions of the adult brain [46], [49]–[51]. Rabbit Map5+ multipolar cells displayed morphological features of astrocytes, molecular profiles of oligodendrocyte precursors [13], [52], and nuclear transcription factors that are expressed by multipotent progenitor cells [53]–[56]. Taken together, such features suggest that these cells fall into the population previously described in rodents and referred to as *synantocytes* (since they form multiple contact with neurons, astrocytes and oligodendrocytes; [13], or as *polydendrocytes* (being related to oligodendrocytes but with more processes and functions; [52]).

Due to the impossibility to localize in the rabbit tissue the Ng2 antigen – a general marker of synantocytes/polydendrocytes in rodents - in spite of the different antibodies employed (see Methods and Table 3), we do not know if the rabbit Map5+ cells represent a subpopulation of Ng2+ cells. Since the number of Map5+ cells in rabbit is lower compared to Ng2+ cells in rodents [13], they could be a newly generated subpopulation of immature progenitors. It is worth noting that a similar population of Map5+ cells could not be detected in the cerebellum of humans [57]. In other words, in the rabbit nervous tissue Map5 seems to function as a marker for young, dividing synantocytes/polydendrocyte-like cells that, due to their spatial distribution and molecular profile, could be a reservoir of proliferating, multipotent progenitor cells within the cerebellum of lagomorphs (see below).

### Dynamics of neurogenic processes in the rabbit cerebellum and persistence of neurogenesis in the adult rabbit cerebellum

The results of the present study show that dynamics of neurogenic processes in the peripuberal and adult rabbit cerebellum are strikingly different from those described in rodents and presently known in mammals (summarized in Figure 8B). In rabbits, the end of cell genesis and the neurochemical maturation of the cerebellum have been fixed around the second postnatal month [58], [59]. Yet, recent studies [9] and the results reported here show that the production of new cell progenitors in the rabbit cerebellum, including neuronal precursors, does not cease after the end of granule cell genesis, but continues at remarkable rates up to and beyond puberty, although progressively decreasing with age. During this period, which is paralleled by the occurrence of a persistent SPL on the cerebellar surface, both GABAergic interneuronal precursors and synantocyte-like cells are generated, whereas a residual genesis of granule cell precursors is restricted to the stage preceding the fourth postnatal month. This fact, in accord with cell fate and positional information provided by transcription factors Pax2, Olig2, and Sox2, suggests that the persistence of both the neuronal and synantocyte/polydendrocytes-like cell populations is not related with the SPL. The transient existence of this layer could be linked to the protracted proliferation and tangential migration of cell precursors in subpial position, which are restricted to the EGL in rodents [60] but find a permissive environment for longer time in lagomorphs.

By extending the countings of BrdU+ nuclei up to 60 days after the last injection we show that most of the cells that remain in the cortex after the first wave of death actually survive for two months (Figure 5). Although cytoplasmic and cytoskeletal markers used to visualize the neuronal cells during the first three-four weeks after their birth subsequently fade and disappear, thus hampering the identification of specific cell shapes (Figure S1B), BrdU/PSA-NCAM double stainings confirm that a consistent population of newlyborn cells maintains traces of PSA-NCAM up to 60 days post-injection survival (Figure S1A). Yet, such type of staining does not provide information about the morphology of these cells, some of which could be dying. For this reason, some BrdU+ cells were detected at the same long term survival by electron microscopy using a pre-embedding technique [61]. Although cytology was hardly detectable, this approach confirmed that healthy cells showing the morphology of both neurons and synantocytes were still in place at that time. The occurrence of synaptic profiles onto the soma of these newly generated cells confirms that they are young neurons establishing contacts with the surrounding cerebellar tissue.

The gradual disappearence of early developmental markers such as PSA-NCAM and DCX can be interpreted as a normal process of cell differentiation in postnatal/adult neurogenic systems [9]–[11], [43]. On the other hand, the absence of general markers of neuronal differentiation and/or specific markers for cerebellar neurons in further steps of cell maturation (see Figure S1B and Table 2) could be a peculiar feature of rabbit cerebellar neurogenesis. Also the expression of the molecule Map5 in newlyborn synantocytes seems to be specific to rabbits, thus confirming that protracted cerebellar neurogenesis in this species can share common rules with other neurogenic systems yet displaying unespected features. For these reasons, further studies are required to unravel the identity and fate of the newly generated cerebellar cells.

The surprising result of the present study consists of evidence that a numerically reduced but still active genesis of both interneuronal and synantocyte-like precursors was detectable in the cerebellar cortex of adult animals, up to three years of age, and even in the absence of a proliferative SPL (Figure 8B). This process was qualitatively similar to that observed at peripuberal stages, involving PSA-NCAM+/DCX+/Pax2+ bipolar- and neuronal-shaped cells in the molecular layer, and Map5+/Olig2+/Sox2+ multipolar cells in all cerebellar layers. Although continuous genesis of glial progenitors and synantocyte-like cells has been described to occur in wide areas of the whole central nervous system (CNS) [12,13; see below], no genesis of neuronal precursors has ever been reported in the adult mammalian cerebellum. The occurrence of newlyborn GABA+ neuronal precursors in the adult rabbit cerebellum suggests that a diluted but widespread genesis of interneurons does occur within the molecular layer of lagomorphs.

Another element of striking novelty in the context of mammalian neurogenesis consists of a protracted capability of rabbit Bergmann glial cells to undergo cell division. The occurrence of the transcription factor Sox2 in the Bergmann glia of rodents [28] has been viewed as a quiescent stem cell potential that cannot be extrinsecated in the highly static cerebellar tissue. On the other hand, such a possibility appears to be real in the environment of the peripuberal/adult rabbit cerebellar cortex.

### The rabbit cerebellar cortex: a permissive tissue for widespread neurogenesis?

Addition of cortical cells in the cerebellar cortex of adult rabbits long time after the complete disappearance of the proliferative SPL clearly rules out the possibility of a superficial source of cell progenitors in the adult. Accordingly, the absence of cell proliferation and markers of neurogenesis within the roof of the fourth ventricle also excludes a periventricular source, thus indicating that all cortical cells born in adult rabbits come from local progenitors. Other elements gathered in the present study converge to this assumption, such as the highly homogeneous distribution of newly generated cells in the whole cerebellar cortex, the persistence of Pax-2 transcription factor in newlyborn cells, namely a landmark for neuronal precursors of neuroepithelial origin during development [8], [17], and the capability of Bergmann glial cells to undergo division.

Local, parenchymal cell progenitors capable of generating neurons have been described to occur within the rabbit caudate nucleus [36]. These cells are independent from the adjacent SVZ and give rise to migrating neuroblasts organized in chains exiting in striatal neurons during adulthood.

The presence of typical bipolar-shaped cells endowed with a cohort of molecules linked to structural plasticity and cell migration in the young, and to a lesser extent, adult rabbit cerebellum does suggest that some of the ex-novo generated cells can also change their position before final differentiation. This aspect appeared to concern mainly the neuronal population, in accord with previous literature referred to other neurogenic systems wherein PSA-NCAM+/DCX+ neuronal precursors are generated following a neurogenic sequence occurring within two-week temporal window [42] (reviewed in [11], [30]). Thus, adult neurogenesis from local parenchymal progenitors could be a feature typical of lagomorphs, involving both brain and cerebellum (see also [42], [62]).

In the context of adult cerebellar neurogenesis, also the multipolar, Map5+ cells could be considered as a novel population of progenitor cells, peculiar to the rabbit cerebellum. Since most of these cells are newly generated and do not populate the CNS as densely as reported for Ng2+ cells in rodents (reviewed in [13]), it is likely that they represent immature cell progenitors trapped in the rabbit cerebellar cortex after pre/post-natal development, that continue to divide locally throughout life. In the rabbit, dividing Bergmann glia could represent the primary source of these progenitor cells, an hypothesis that is strengthened by the fact that all these cells express the transcription factor Sox2, and the multipolar cells also express Olig2. Sox2 is implicated in the proliferation/maintenance of neural stem cells and in adult neurogenesis [53]–[55]. In particular, it is important in maintaining embryonic stem cells in a pluripotent state that actively inhibits neuronal differentiation in neural precursor cells [63]. Also Olig2 is involved in development of neuronal and glial precursors, and continues to be expressed in multipotent progenitor cells of the adult CNS [64], although its role remains obscure (reviewed in [65]). Thus, the presence of these transcription factors suggests that multipolar Map5+ cells could be multipotent progenitors instead of simple glial progenitors.

### Possible functional implications and future perspectives

An explanation for differences in neurogenic potential between rodents and lagomorphs could reside in the pronounced variation in life span among these animal species, influencing distinct patterns of growth, maturation, and senescence that may have an impact on the extent and plasticity of neurogenesis throughout life (see [30], [34]). In this context, tissue growth may be a critical feature for the regulation of adult neurogenesis, what could explain why teleost fish species that have indeterminate growth also have continual addition of new neurons throughout life and striking capacity for brain repair and regeneration [1].

This perspective leaves open the intriguing possibility that an undetected, delayed genesis of neuronal and glial progenitor cells could exist in other mammalian species characterized by slow growth and long lifespan, including primates and humans. It is known that brain neurogenic sites exist in these species [66], [67], but their activity declines with age [68] even more dramatically than in rodents [69]. Furthermore, it is believed that what really hampers the self-repair of adult mammalian brains might be linked to their failure in allowing the proliferation/migration/differentiation of parenchymal progenitor cells rather than a lack of progenitors [70]. Uncovering whether the potentialities proper of the rabbit CNS do exist in young humans could be of paramount importance in the perspective of modulating widespread adult neurogenesis, beyond the highly restricted - periventricular and hippocampal - neurogenic zones. In this context, the rabbit brain and cerebellum could be a suitable model for studying nervous tissue permissiveness in mammals.

## Materials and Methods

### BrdU injections and tissue preparation

Experimentation was conducted in accordance with current EU and Italian laws (Italian Ministry of Health, authorization n. 66/99-A). Three postnatal (P 10), twentyeight peri-puberal (3 month-old: n = 22; 4 month-old: n = 2; 5 month-old: n = 2; 6 month-old: n = 2), and six adult (1 year-old: n = 3; 3 year-old: n = 3) New Zealand White rabbits (Orictolagus cuniculus, Charles River, Milan, I) were used for light/confocal microscopy. Sixteen animals (3 month-old) received intraperitoneal injections of BrdU (Sigma; 40 mg/Kg). Three received a single injection and were killed after 2 hours (h). Thirteen rabbits received one daily injection for 5 days and were killed 2 h, 5, 10, 30, 60 days (n = 3 each survival time, except for 5 days: n = 1) after the last injection. All adult rabbits received one daily injection for 5 days and were killed after 10 days after the last injection. In addition, four peri-puberal (three month-old) rabbits that received one daily injection of BrdU for 15 days, and were killed after two months since the first injection, were used for electron microscopy (a summary of all animals employed in this study is given in Figure S3).

Animals were anaesthetized with a ketamine/xylazine solution (100 mg/Kg body weight: 33 mg/Kg body weight) and perfused as previously described [35], [9], [71]. Cerebella were extracted carefully to preserve the pia mater, post-fixed 6 h (light microscopy), then the hemicerebella separated. All postnatal, all adult, and twentysix peri-puberal cerebella, were frozen at −80°C and cryostat (25 µm thick) sectioned in series, whereas three peri-puberal cerebella were sectioned with a vibratome (100 µm thick). According to the aims of different experiments, cerebella were cut along sagittal and coronal orientation.

### Histological procedures and immunohistochemistry

Immunohistochemical reactions were carried out by using single peroxidase and double immunofluorescence methods on cryostat sections incubated overnight at room temperature with primary antibodies (Table 3). All polyclonal antisera used did not give any problem or aspecificity on the rabbit cerebellar tissue.

Immunohistochemistry for singles stainings was revealed using indirect peroxidase techniques with biotinylated secondary antibodies (anti-goat IgG, anti-mouse IgM, anti-rabbit IgG, Vector Labs, Peterbourough, UK; mouse IgG, Sigma, Saint Louis, USA), treated with an avidin-biotin complex (Vector Elite kit; Vector Labs; Peterbourough, UK) and detected with 3,3'diaminobenzidine (DAB) in TRIS-HCl 50 mM pH 7,6, containing 0,025% hydrogen peroxide for 3 minutes then washed in TRIS-HCl 50 mM pH 7.6.

For double staining indirect immunofluorescence procedures using FITC-avidin (Vector Labs)+Cy3 conjugated antibodies (anti-mouse IgG+IgM; anti-Rabbit IgG Jackson Immunoresearch Laboratories, West Grove, USA) were used. Antibodies were diluted in 0,01 M PBS, pH 7,4, containing 0,5% Triton X-100. Fluorescent specimens mounted in Mowiol 4–88 (Calbiochem-EMD Chemicals, Darmstadt, D) were observed with a laser scanning Olympus Fluoview confocal system.

Cells undergoing apoptotic cell death were detected by TUNEL analysis using the Apop-Tag Kit (Chemicon) according to the supplier's instructions. Microglial cells were detected with Fluorescein Griffonia (Bandeiraea) Simplicifolia lectin 1, Isolectin B4 1/200 (FL-1201 Vector Laboratories, Burlingame, USA).

### Pre-embedding immunolectron microscopy

The ultrastructural localization of exogenously-administered BrdU was obtained employing a pre-embedding immunoperoxidase reaction [61]. Rabbits were perfused with 4% paraformaldehyde and picric acid 1% in 0,1 M sodium phosphate buffer (PBS), postfixed 6 h at 4°C, washed overnight in cold PBS, and vibratome sectioned (70 µm thick). After aldehydes blocking in 100 mM NH_4_Cl for 2 h at room temperature (RT), slices were washed in PBS, pre-treated with 2 M HCl for 15 min at 37°C, neutralized for 10 min in buffer at pH 8,4 and subsequently incubated in 1% NGS + 0,1% Triton X-100 in PBS for 1 h at 4°C and then in anti-BrdU diluted 1∶500 in PBS overnight at RT. After washing in PBS, slices were incubated in biotinylated anti-rat IgG (Vector labs) diluted 1∶200 for 2 h. Sections were subsequently washed and treated with an avidin-biotin complex (Vector Elite kit; Vector Labs) for 1 h at RT. After washing, sections were processed for visualization with 0.2% diaminobenzidine (Sigma, Saint Louis, USA) in TRIS-HCl 50 mM pH 7,6, containing 0,025% hydrogen peroxide for approximately 3 minutes then washed in TRIS-HCl 50 mM pH 7,6. Small pieces of the molecular layer containing single BrdU+ nuclei not associated with blood vessels were dissected under a steromicroscope, washed in PBS, postfixed in 2% Osmium Tetroxide and FeCN 3% for 1 h at 4°C, washed overnight with maleate buffer, counterstained in uranyl acetate 1%, washed with maleate buffer, dehydrated and embedded in araldite as previously described [71]. Semi-thin sections (1 µm thick) stained with 1% toluidine blue and 0,5% NaHCO_3_, were cut sequentially and observed in light microscopy until identification of the BrdU-marked nuclei. Starting from this point, ultra-thin sections were harvested and examined with a Philips CM10 transmission electron microscope.

### Quantitative analyses

Countings of PSA-NCAM+ and Map5+ cells have been performed at different ages (3, 6, 12, and 36 months for PSA-NCAM; 3 and 36 months for Map5; n = 3, each age considered except for 6 months n = 2). The analysis has been conducted on six representative parasagittal cryostat sections (cut at different medial-lateral levels) from each animal. The cells present in each layer (molecular layer, granule cell layer, and white matter axis of the lamellae) were counted, and results were expressed as number of cells/10 mm of pial surface (in a total surface of 275 mm; see Table 1). The number of different PSA-NCAM+ morphological types was also evaluated and expressed as percentages in Figure 1R. Cells present in the SPL, due to the presence of chains, were extrapolated by considering 2 bipolar cells/chain as previously described [9]. The number of PSA-NCAM and Map 5 + cells, in each layer and at different post-natal ages (PSA-NCAM: 3 month-old vs 6 month-old and 3 month-old vs adult; MAP5: 3 month-old vs adult) has been analyzed with Student's Test- T (Primer statistical software for Windows). The number of PSA-NCAM and Map5+ cells, in different layers (ML vs GL) at the same post-natal age, has been analyzed with Student's Test- T.

Finally the total number of PSA-NCAM+ vs Map5+ cells has been analyzed in 3 month-old and adult animals with Student's Test- T. A value of p ≤ 0.05 was accepted as statistically significant.

Quantitative analysis of BrdU+ cells was performed in nine 3 month-old rabbits that received 5 daily injections, and were subsequently killed after 2 h (n = 3), 10 (n = 3) and 60 (n = 3) days after the last injection. These countings were carried out on cortical tissue corresponding to a total of 200 mm pial surface in three representative parasagittal cryostat sections (cut at different medial-lateral levels; based on the fact that the newly generated cells were distributed homogeneously in the entire cortical extension; see Results) taken from cerebella of each animal (see [9]). The BrdU-immunoreactive nuclei present on a single focal plane of the molecular and granule cell layer were counted. Nuclei in the SPL (5 µm beneath the pial surface) have not been taken into account.

The number of BrdU+ cells has been analysed by one-way ANOVA (Primer statistical software for Windows) with main factor time (post-injection survival times). Post-hoc comparisons were carried out with Tukey HSD test.

The number of Brdu+ cells in each cerebellar layer at the different survival time has been analysed with Student's test T. A value of p≤0.05 was accepted as statistically significant

The amount of apoptotic cell death was analysed by counting TUNEL-labelled cells in the cerebellum of 3, 4 and 36 months old animals (n = 3). Immunoreactive nuclei present on a single focal plane of the SPL and cerebellar cortical layers were counted in three representative sections, and expressed as number of cells/10 mm of pial surface (in a total surface of 275 mm; see Table 1). The number of TUNEL+ cells at the different postnatal ages has been analysed with Student's test T. A value of p≤0.05 was accepted as statistically significant.

The percentage of double labelled cells versus BrdU+ cells was evaluated for PSA-NCAM, Pax2, Map5, Sox2. These countings were carried out on three animals for each BrdU treatment (see above). Cortical tissue corresponding to a total of 200 mm pial surface in six representative parasagittal cryostat sections (cut at different medial-lateral levels) taken from cerebella of each animal.

The percentage of bipolar and intermediate or neuronal-like BrdU/PSA-NCAM double labelled cells was then compared between 5 and 15 days after BrdU injection. These countings were performed as the other double labelling countings. The data were analysed with Student's Test T. A value of p≤0.05 was accepted as statistically significant.

## Supporting Information

Figure S1 Analysis of newly generated cells at long term survival time. A, Percentage of BrdU+/PSA-NCAM+ and BrdU+/Map5+ double-stained cells on the total BrdU+ cells at different survival times. B, Time course of cytoplasmic marker expression (PSA-NCAM, doublecortin and Map5) in newly generated cells of the peripuberal/adult rabbit cerebellar cortex. PSA-NCAM and doublecortin start to fade at the end of the first month after their genesis, whereas Map5 appears during the second week. Brdu+ nuclei (black dots) that are not associated with these markers mostly belong to the same cell populations at early (multipolar cells) or at later stages (polarized, neuronal cells) of their differentiation. C,D, A smaller population of newly generated cells involves glial elements: C, rare BrdU+ nuclei can correspond to dividing microglial cells (here, a cell in the molecular layer visualized 2 months after BrdU injection); D, Cell divisions in a BLBP+ Bergmann glial cell (here during the first 5 days after BrdU treatment).
(1.19 MB TIF)

Figure S2 Quantitative analyses on single and double stainings. A, BrdU+/PSA-NCAM+ cells with different morphologies. The number of double labelled bipolar cells significantly decreases from 5 to 15 days after BrdU treatment (5 days: 34,56 +/− 3,41%; 15 days: 22,90 +/− 2,28%, p = 0,008), whereas the number of intermediate-shaped and neuronal-like cells significantly increases (5 days: 13,75 +/− 3,17%; 15 days: 32,50 +/− 5,99%, p = 0,010). B, Percentage of BrdU+/Pax2+ double labelled cells with respect to total BrdU+ cells at different survival times (5 days: 20,02 +/− 2,03%; 15 days: 39,48 +/− 5,16%; 65 days: 1,76 +/− 1,07%). C, Percentage of BrdU+/Sox2+ double labelled cells with respect to total BrdU+ cells at different survival times (15 days: 64,71 +/− 2,71%; 65 days: 76,98 +/− 0,79%).
(0.20 MB TIF)

Figure S3 Animals used in this study.
(0.04 MB DOC)
