# Supplementary figures and images for: Genesis of Neuronal and Glial Progenitors in the Cerebellar Cortex of Peripuberal and Adult Rabbits
Source: PLoS One. 2008 Jun 4;3(6):e2366. doi: 10.1371/journal.pone.0002366 (PMC2396292; doi:10.1371/journal.pone.0002366)

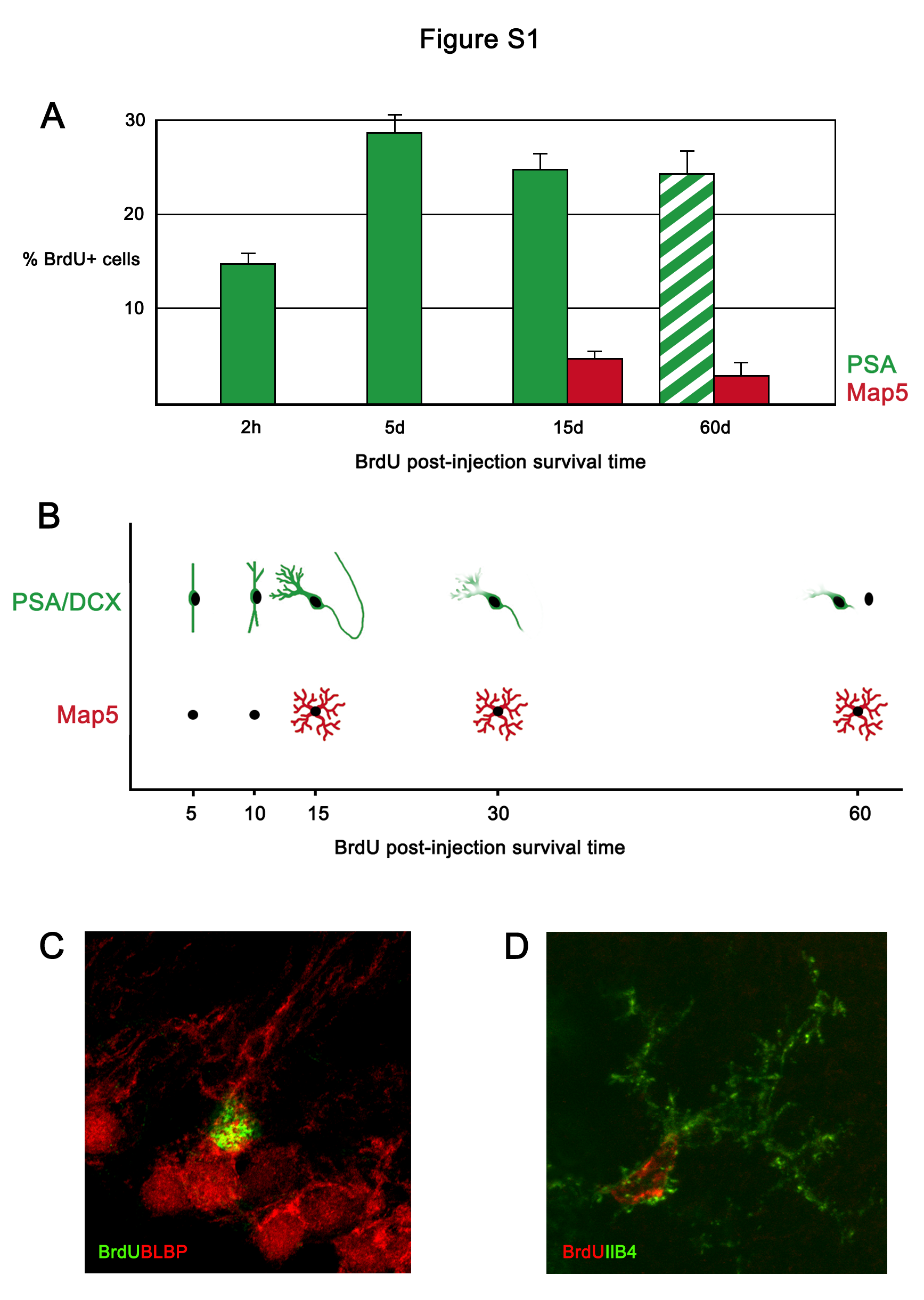

Supplement: Figure S1 — Analysis of newly generated cells at long term survival time. A, Percentage of BrdU+/PSA-NCAM+ and BrdU+/Map5+ double-stained cells on the total BrdU+ cells at different survival times. B, Time course of cytoplasmic marker expression (PSA-NCAM, doublecortin and Map5) in newly generated cells of the peripuberal/adult rabbit cerebellar cortex. PSA-NCAM and doublecortin start to fade at the end of the first month after their genesis, whereas Map5 appears during the second week. Brdu+ nuclei (black dots) that are not associated with these markers mostly belong to the same cell populations at early (multipolar cells) or at later stages (polarized, neuronal cells) of their differentiation. C,D, A smaller population of newly generated cells involves glial elements: C, rare BrdU+ nuclei can correspond to dividing microglial cells (here, a cell in the molecular layer visualized 2 months after BrdU injection); D, Cell divisions in a BLBP+ Bergmann glial cell (here during the first 5 days after BrdU treatment). (1.19 MB TIF) [file pone.0002366.s001.tif]

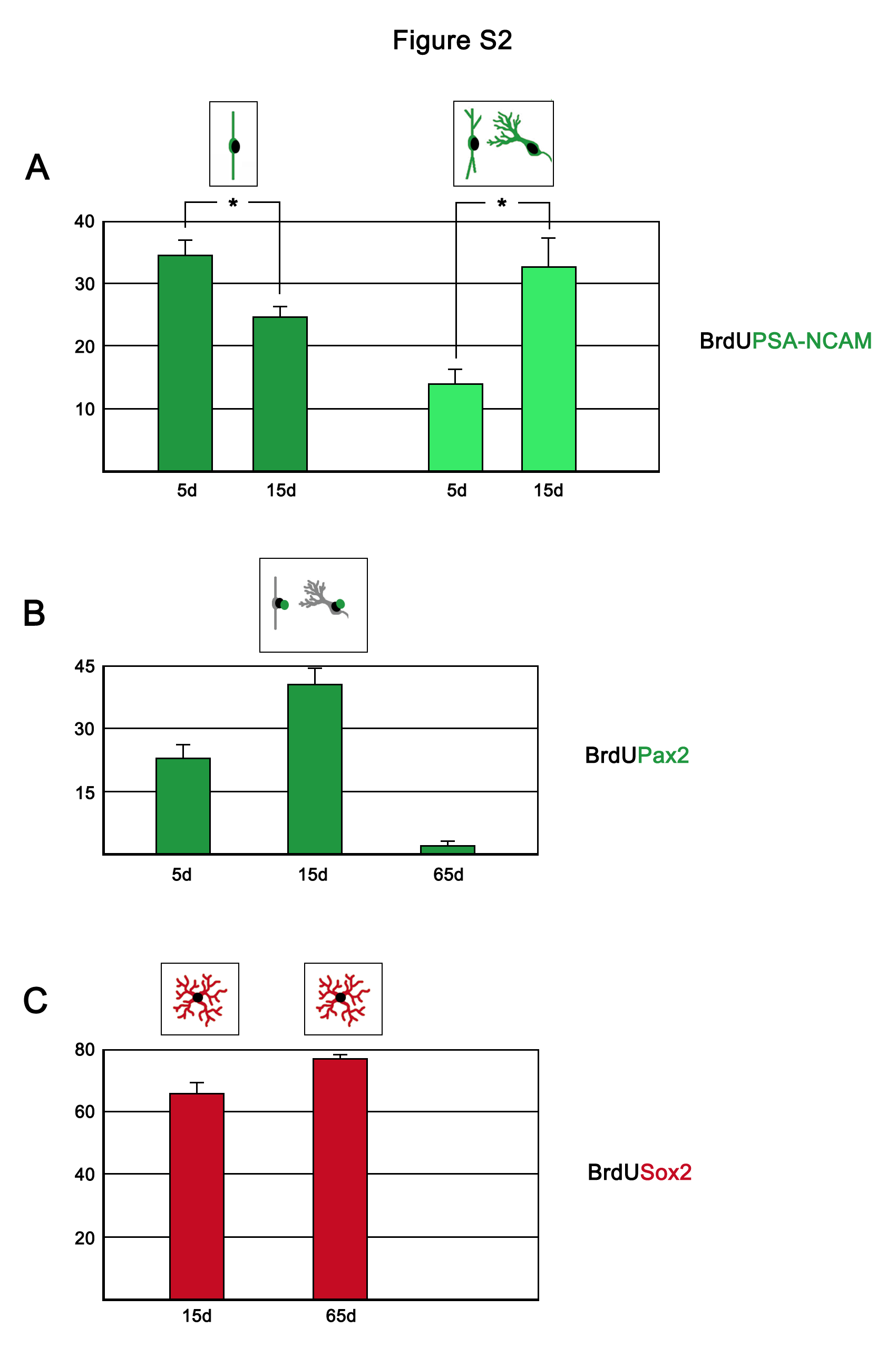

Supplement: Figure S2 — Quantitative analyses on single and double stainings. A, BrdU+/PSA-NCAM+ cells with different morphologies. The number of double labelled bipolar cells significantly decreases from 5 to 15 days after BrdU treatment (5 days: 34,56 +/− 3,41%; 15 days: 22,90 +/− 2,28%, p = 0,008), whereas the number of intermediate-shaped and neuronal-like cells significantly increases (5 days: 13,75 +/− 3,17%; 15 days: 32,50 +/− 5,99%, p = 0,010). B, Percentage of BrdU+/Pax2+ double labelled cells with respect to total BrdU+ cells at different survival times (5 days: 20,02 +/− 2,03%; 15 days: 39,48 +/− 5,16%; 65 days: 1,76 +/− 1,07%). C, Percentage of BrdU+/Sox2+ double labelled cells with respect to total BrdU+ cells at different survival times (15 days: 64,71 +/− 2,71%; 65 days: 76,98 +/− 0,79%). (0.20 MB TIF) [file pone.0002366.s002.tif]
