# Supplementary material for: Genesis of Neuronal and Glial Progenitors in the Cerebellar Cortex of Peripuberal and Adult Rabbits
Source: PLoS One. 2008 Jun 4;3(6):e2366. doi: 10.1371/journal.pone.0002366 (PMC2396292; doi:10.1371/journal.pone.0002366)
Supplement: Figure S3 — Animals used in this study. (0.04 MB DOC) [file pone.0002366.s003.doc]

# Figure S3

| **Animal** | **Age** | **Treatment** | **Survival *** | Analyses |
| --- | --- | --- | --- | --- |
| 1-3 | P 10 | - | - | Immunohistochemistry |
| 4-6 | 3 month-old | 1 BrdU injection | 2 hours | Quantitative analysis of BrdU+/PSA-NCAM+ and BrdU+/Map5+ double labelled cells |
| 7-9 | 3 month-old | 1 daily BrdU injection for 5 days | 2 hours | Quantitative analysis of BrdU+ cells, BrdU+/PSA-NCAM+, BrdU+/Map5+, BrdU+/Pax2+, double labelled cells |
| 10 | 3 month-old | 1 daily BrdU injection for 5 days | 5 days | Qualitative analysis of BrdU+/PSA-NCAM+ cells |
| 11-13 | 3 month-old | 1 daily BrdU injection for 5 days | 10 days | Quantitative analysis of BrdU+ cells, BrdU+/PSA-NCAM+, BrdU+/Map5+, BrdU+/Pax2+, double labelled cells |
| 14-16 | 3 month-old | 1 daily BrdU injection for 5 days | 30 days | Quantitative analysis of BrdU+/Pax2+ double labelled cells |
| 17-19 | 3 month-old | 1 daily BrdU injection for 5 days | 60 days | Quantitative analysis of BrdU+ cells BrdU+/Map5+, BrdU+/Pax2+ and BrdU+/IlB4+ double labelled cells |
| 20-24 | 3 month-old | 1 daily BrdU injection for 15 days | 45 days | Immunoelectron microscopy |
| 25-27 | 3 month-old | - | - | Quantitative analysis of TUNEL+, PSA-NCAM+, MAP5+cells; immunohistochemistry |
| 28-30 | 3 month-old | - | - | Vibratome sectioned for immunohistochemistry |
| 31-32 | 4 month-old | - | - | Quantitative analysis of TUNEL+, PSA-NCAM+cells; immunohistochemistry |
| 33-34 | 5 month-old | - | - | Immunohistochemistry |
| 35-36 | 6 month-old | - | - | Quantitative analysis of PSA-NCAM+cells; immunohistochemistry |
| 37-39 | 1 year-old | 1 daily BrdU injection for 5 days | 10 days | Quantitative analysis of PSA-NCAM+cells; immunohistochemistry |
| 40-43 | 3 year-old | 1 daily BrdU injection for 5 days | 10 days | Quantitative analysis of TUNEL+, PSA-NCAM+, MAP5+cells; immunohistochemistry |

(*) After the last BrdU injection.

| **Animal** | **Age** | **Treatment** | **Survival** | **Analyses** |
| --- | --- | --- | --- | --- |
| 1-3 | P 10 | - | - | immunohistochemistry |
| 4-6 | 3 month-old | 1 BrdU injection | 2 hours | Quantitative analysis of BrdU+/PSA-NCAM+ and BrdU+/Map5+ double labelled cells |
| 7-9 | 3 month-old | 1 daily BrdU injection for 5 days | 2 hours | Quantitative analysis of BrdU+ cells, BrdU+/PSA-NCAM+, BrdU+/Map5+, BrdU+/Pax2+, double labelled cells |
| 10 | 3 month-old | 1 daily BrdU injection for 5 days | 5 days | Qualitative analysis of BrdU+/PSA-NCAM+ cells |
| 11-13 | 3 month-old | 1 daily BrdU injection for 5 days | 10 days | Quantitative analysis of BrdU+ cells, BrdU+/PSA-NCAM+, BrdU+/Map5+, BrdU+/Pax2+, double labelled cells |
| 14-16 | 3 month-old | 1 daily BrdU injection for 5 days | 30 days | Quantitative analysis of BrdU+/Pax2+ double labelled cells |
| 17-19 | 3 month-old | 1 daily BrdU injection for 5 days | 60 days | Quantitative analysis of BrdU+ cells BrdU+/Map5+, BrdU+/Pax2+ and BrdU+/IlB4+ double labelled cells |
| 20-23 | 3 month-old | 1 daily BrdU injection for 15 days | 45 days | Immunoelectron microscopy |
| 24-26 | 3 month-old | - | - | Quantitative analysis of TUNEL+, PSA-NCAM+, MAP5+cells; immunohistochemistry |
| 27-29 | 3 month-old | - | - | Vibratome sectioned for immunohistochemistry |
| 30 | 3 month-old | - | - | Immunohistochemistry |
| 31-33 | 4 month-old | - | - | Quantitative analysis of TUNEL+, PSA-NCAM+cells; immunohistochemistry |
| 34-35 | 5 month-old | - | - | Immunohistochemistry |
| 36-37 | 6 month-old | - | - | Quantitative analysis of PSA-NCAM+cells; immunohistochemistry |
| 38-40 | 1 year-old | 1 daily BrdU injection for 5 days | 10 days | Quantitative analysis of PSA-NCAM+cells; immunohistochemistry |
| 41-43 | 2 year-old | - | - | Quantitative analysis of PSA-NCAM+cells; immunohistochemistry |
| 44-46 | 3 year-old | 1 daily BrdU injection for 5 days | 10 days | Quantitative analysis of TUNEL+, PSA-NCAM+, MAP5+cells; immunohistochemistry |

Gli animali di 6 mesi erano 2 nella versione già sottomessa. Su questi animali però abbiamo fatto la quantificazione delle cellule PSA nei vari strati.
